# Integration of resolving topological indices and ADMET analysis with MCDM methods (TOPSIS and SAW) for ranking breast cancer therapeutics

**DOI:** 10.3389/fbinf.2026.1913227

**Published:** 2026-10-14

**Authors:** E. Pandeeswari, J. Ravi Sankar

**Affiliations:** Department of Mathematics, School of Advanced Sciences, Vellore Institute of Technology, Vellore, Tamil Nadu, India

**Keywords:** ADMET properties, breast cancer drugs, Multi-Criteria Decision-Making, QSPR analyses, resolving topological indices

## Abstract

Recent studies have established a relationship between the topological structure of chemical compounds and their pharmacokinetic properties. These properties, such as Absorption, Distribution, Metabolism, Excretion, and Toxicity (ADMET) parameters, are essential for evaluating drugs. In this study, 15 breast cancer drug molecules, namely, tamoxifen, elacestrant, fulvestrant, toremifene, megestrol acetate, anastrozole, exemestane, letrozole, alpelisib, ixabepilone, abemaciclib, capivasertib, ribociclib, neratinib, and olaparib, were transformed into molecular graphs; $M_{\beta }$ -polynomials were used to calculate resolving topological indices. The study utilizes resolving topological indices to characterize the molecular structures of breast cancer drugs and formulates Quantitative Structure–Property Relationship (QSPR) models to forecast their ADMET properties. It involves the computation of resolving topological indices, conducting regression analyses to determine significant indices, and applying these indices in multicriteria decision-making methods to rank the drugs. The drugs were then ranked using decision-making approaches, especially Technique for Order Preference by Similarity to Ideal Solution (TOPSIS) and Simple Additive Weighting (SAW), using the computed indices and ADMET data. Based on the model findings, fulvestrant, neratinib, and ribociclib were ranked highest, whereas letrozole and exemestane were placed lowest. However, this ranking cannot accurately represent clinical outcomes and is only based on the mathematical model outputs. The consistency of the study was supported by a comparison plot that showed strong agreement between the two approaches. This study demonstrates how a systematic and quantitative framework for evaluating and ranking drug candidates may be obtained by merging chemical graph theory with decision-making techniques. This methodology may be extended to bigger datasets in future studies.

## 1 Introduction

Breast cancer is one of the most frequent and difficult malignancies affecting women globally. Its therapy is based on various techniques that target molecular pathways necessary for tumor development and dissemination. Food and Drug Administration-approved drugs act by inhibiting hormone synthesis, affecting critical physiological processes, changing signaling pathways, or exploiting DNA repair flaws. Understanding these pathways helps to improve therapy and results. A tailored, integrated strategy is required to properly manage this complex disease and enhance patient outcomes.

Chemical graph theory analyzes molecular structures using graph-theoretical approaches to determine topological indices that characterize structural attributes, including size, branching, and connection. To connect molecular structure to physicochemical and biological features, these indices are often employed in QSPR research (Gonzalez-Diaz et al., 2007; George, 1997).

Slater presented the concept of metric dimension, which is based on a resolving set (Slater, 1975). Harary and Melter (1976) explored it separately. A subset S⊆V is said to be a resolving set (or locating set) if there exists at least one vertex x∈S such that d(x,a)≠d(x,b) . The metric dimension of a graph is G , the lowest cardinality of its resolving set, represented as β(G) . A metric basis of G is a resolving set of size β(G) . Our earlier research demonstrated the efficacy of metric and edge metric dimensions as molecular descriptors for assessing structural uniqueness and drug-related features (Pandeeswari and Ravi Sankar, 2025a). In the present study, we use brute-force algorithm 1 to find the resolving sets and calculate the metric dimension of each molecular graph studied. This approach requires less time and produces efficient results for the graphs under consideration. The M-polynomial developed by Deutsch and Klavžar (2014) makes it easier to compute degree-based topological indices in chemical graph theory. This program effectively calculates indices that are essential for comprehending molecular characteristics, such as Randic and Zagreb. The COVID-19 pandemic underlined the necessity for fast drug development utilizing computational technologies such as QSPR and resolving polynomial graph theory. Degree-based topological indices and a two-variable resolving polynomial were used to examine and estimate the properties of COVID-19 medicines (Chandrakala et al., 2024). In our study, we applied the resolving polynomial approach to FDA-approved breast cancer drugs and derived resolving-degree-based topological indices to analyze their molecular structures.

To address this, a recent study has proposed the notion of resolving topological indices (RTIs), which take into consideration a graph’s resolving set and metric dimension while computing degree-based descriptors. The resolving degree of a vertex indicates its contribution to uniquely identifying other vertices in the structure, thus providing a new level of structural knowledge. In their pioneering work, researchers such as Chandrakala et al. (2024) used this method to diverse chemicals and nanostructures, indicating that resolving-based indices may improve QSPR and Quantitative Structure-Activity Relationship (QSAR) models. For example, research has found that combining the resolving polynomial framework with traditional topological indices enhances the prediction of molecular characteristics for antiviral drugs, silicate networks, and nanostructures.

Recent studies have shown that topological and graph-theoretical descriptors, including degcity indices, Wiener indices (Kour and Sankar, 2024; Kour and Sankar, 2025) and graph energy are excellent at predicting molecular features and assessing structural aspects of chemical and algebraic systems (Pandeeswari and Sankar J, 2025; Clement Johnson RayerRavi Sankar Jeyaraj, 2023). The technical efficiency of decision-making units (DMUs) was investigated using data-driven modeling tools to discover productivity gaps and improve decision-making accuracy in complex analytical systems (Venkateswarlu et al., 2018). Mahaboob et al. (2017) and Sankar and Felix (2016) used an updated fuzzy DEMATEL approach to examine complicated interrelationships among criteria, thereby improving decision-making precision and reliability in multi-criteria assessment systems. Narayana et al. (2018a) and Narayana et al. (2018b) focused on resolving model misspecification and selection issues in stochastic linear regression frameworks, improving prediction accuracy, resilience, and overall reliability of statistical model evaluation.

Operations research focuses on creating and using advanced methods of analysis to enhance decision-making. The operations research field of MCDM (Multi-Criteria Decision-Making) is outstanding. According to one viewpoint, mathematical programming has emerged as a powerful tool for analyzing numerous intentions and determining the optimal course of action. When several criteria (or objectives) must be taken into account simultaneously in order to rank or select among alternatives (in this case, drugs), MCDM is about making decisions. Researchers enhance the optimization of process parameters in turning processes through MCDM techniques. These approaches can be categorized based on decision-making frameworks and linear and non-linear modeling structures.

Some MCDM approaches include Technique for Order Preference by Similarity to Ideal Solution (TOPSIS), Simple Additive Weighting (SAW), analytical-network process (ANP), and analytical hierarchy process (AHP). MCDM approaches were used to optimize drug delivery vehicles (Omoyayi et al., 2024). Asthma treatment drugs were optimized using MCDM techniques (Sultana, 2024). To rank drugs against cancer, topological indices were used for the MDCM method (Preetha Pandi et al., 2024). The top rating for the MCDM-based development of renewable energy sources was given by Kumar et al. (2017).

Recent studies have employed graph-theoretic descriptors, QSPR analysis, and ranking methods to assess drug characteristics and therapeutic performance in diverse disease areas (Ahmed et al., 2026; Iqbal et al., 2026; Waheed Rasheed et al., 2026). Our study examined 15 important breast cancer drugs, and their molecular structures are shown in Figure 1, which include tamoxifen, elacestrant, toremifene, fulvestrant, megestrol acetate, anastrozole, exemestane, letrozole, alpelisib, ixabepilone, abemaciclib, capivasertib, ribociclib, neratinib, and olaparib. Table 1 contains thorough information about these drugs. To assess their overall drug likeness and therapeutic potential, key ADMET properties water solubility (WS), Caco-2 permeability, skin permeability (SP), oral acute toxicity (ORAT), and oral chronic toxicity (ORCT) were analyzed alongside relevant topological indices to establish meaningful QSPR relationships. Finally, a multicriteria decision-making (MCDM) technique was used to rank these therapeutic candidates systematically including the estimated weights and normalized values to determine the most promising molecules.

**FIGURE 1:**
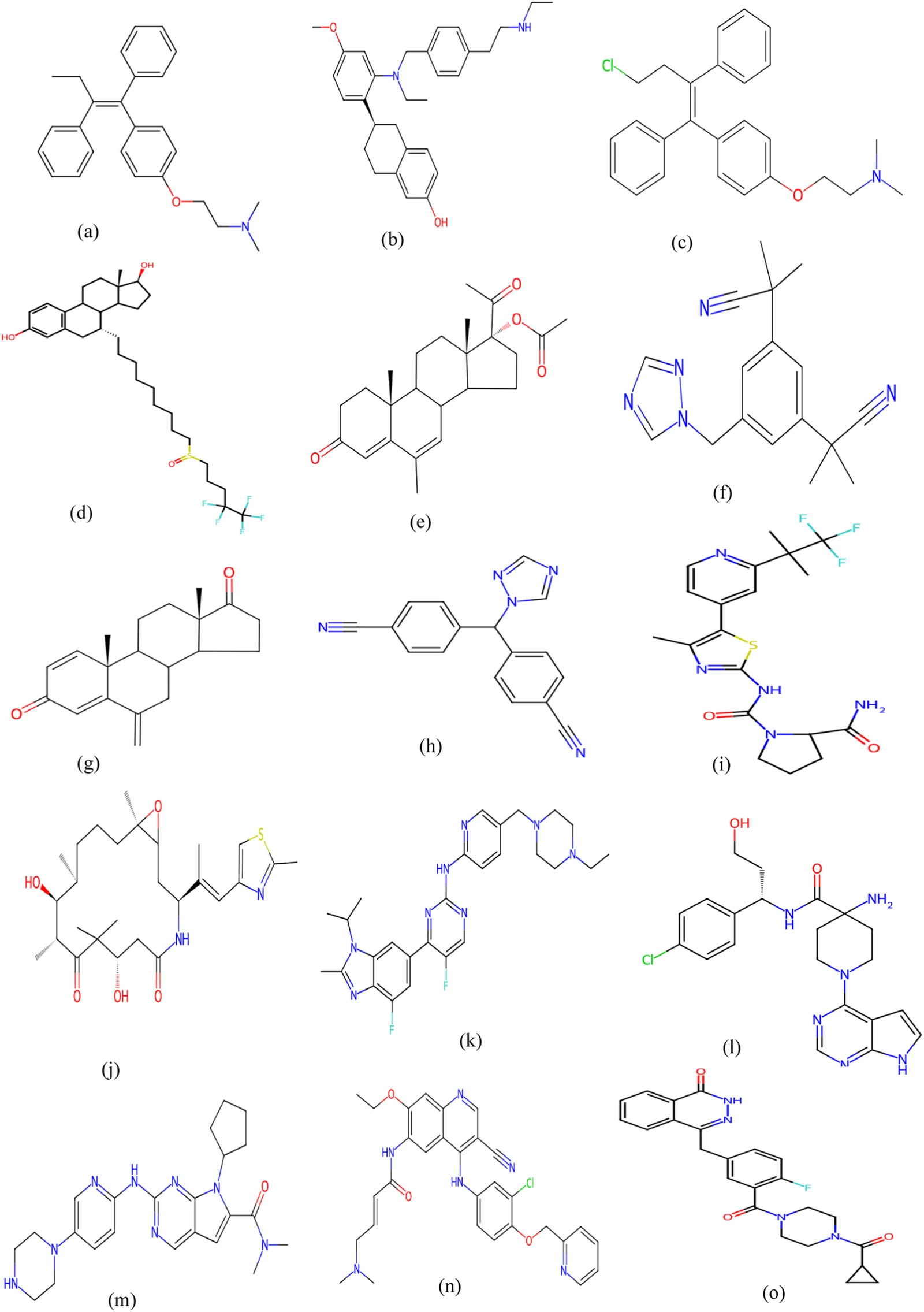
**(a)** Tamoxifen, **(b)** Elacestrant, **(c)** Toremifene, **(d)** Fulvestrant, **(e)** Megestrol Acetate, **(f)** Anastrozole, **(g)** Exemestane, **(h)** Letrozole, **(i)** Alpelisib, **(j)** Ixabepilone, **(k)** Abemaciclib, **(l)** Capivasertib, **(m)** Ribociclib, **(n)** Neratinib, **(o)** Olaparib.

**TABLE 1:** Drugs against breast cancer and mechanism of action.

Mode of action	Drug	Mechanism of action
Hormone modulators	Tamoxifen	Blocks estrogen from binding its receptor, lowers free estradiol by increasing SHBG, and induces apoptosis in estrogen receptor (ER)-positive cells
	Elacestrant	Binds to estrogen receptor alpha (ER α ) and inhibits it and promotes the destruction of (ER α )
	Fulvestrant	Disrupts the estrogen receptor, resulting in degradation of the receptor complex and impaired receptor binding to estrogen
	Toremifene	Binds to estrogen receptors and may have estrogenic and anti-estrogenic effects; its antitumor effect is possibly related to its antiestrogenic effect
	Megestrol acetate	Mechanism of action not well defined, may be a progesterone receptor agonist, and may slow the growth of tumors
Aromatase inhibitors	Anastrozole	Selective and competitive blockage of the aromatase enzyme
	Exemestane	Irreversible inhibitor of the aromatase enzyme
	Letrozole	A non-steroidal inhibitor which competitively binds to the active site of the aromatase enzyme (CYP19A1)
Microtubule inhibitors	Ixabepilone	Attaches to and stabilizes the microtubules so that no cell division can occur
Kinase inhibitors	Alpelisib	Selectively inhibits PI3Kα , a subunit of phosphatidylinositol 3-kinase, which is used by cancer cells to grow
	Abemaciclib	Inhibits activities of CDK4/6, preventing phosphorylation of Rb causing cell-cycle arrest in the G1 phase
	Capivasertib	Inhibits all three isoforms of AKT (AKT1, AKT2, and AKT3), which is a key pathway for cell growth and survival
	Ribociclib	Blocks the action of proteins known as cyclin-dependent kinases CDK4 and CDK6, which prevent uncontrolled cell growth
	Neratinib	This drug irreversibly inhibits EGFR, HER2 and HER4 receptors—all vital to cancer cell growth—and downstream activating pathways
PARP inhibitors	Olaparib	A potent and specific inhibitor of the PARP1 and PARP2 enzymes that stops cancer cells from repairing their DNA.

Resolving topological indices offer an alternate structural description of molecular graphs to standard topological indices and molecular descriptors. These indices rely on a molecular graph’s metric structure and employ resolving sets and degrees to identify vertices based on their structural locations. This extra structural information could help identify molecular properties that are not fully captured by standard descriptors.

Although numerous QSPR studies have used classical topological indices to predict molecular properties, the current study presents a novel framework that employs resolving topological indices generated from the $M_{\beta }$ -polynomial. Although previous studies have mainly focused on property prediction (Pandeeswari and Ravi Sankar, 2025b), this work combines resolving topological indices with ADMET properties and uses MCDM approaches (TOPSIS and SAW) to systematically rank breast cancer drugs. This integrated mathematical and decision-making framework offers a complete method for assessing drug candidates based on both structural and pharmacokinetic properties. In this study, two major procedures are taken: first, the resolving topological indices are computed, and second, a ranking methodology is performed utilizing the multicriteria decision-making (MCDM) method. Each descriptor is additive; therefore, the results are achieved by adding the computed terms. The indices β $M_{1}\left(G\right)$ , β $M_{1}^{*}\left(G\right)$ , β $M_{2}\left(G\right)$ , β HM(G) , and β F(G) . Python is used to carefully validate all computations. The pkCSM database contains experimental or computational data on breast cancer treatments. The obtained resolving topological indices and experimental or computational data are then combined with MCDM methods, especially TOPSIS and SAW, to rank the drugs. These observations are processed and evaluated using MS Excel. Figure 2 illustrates the workflow of the proposed methodology.

**FIGURE 2:**
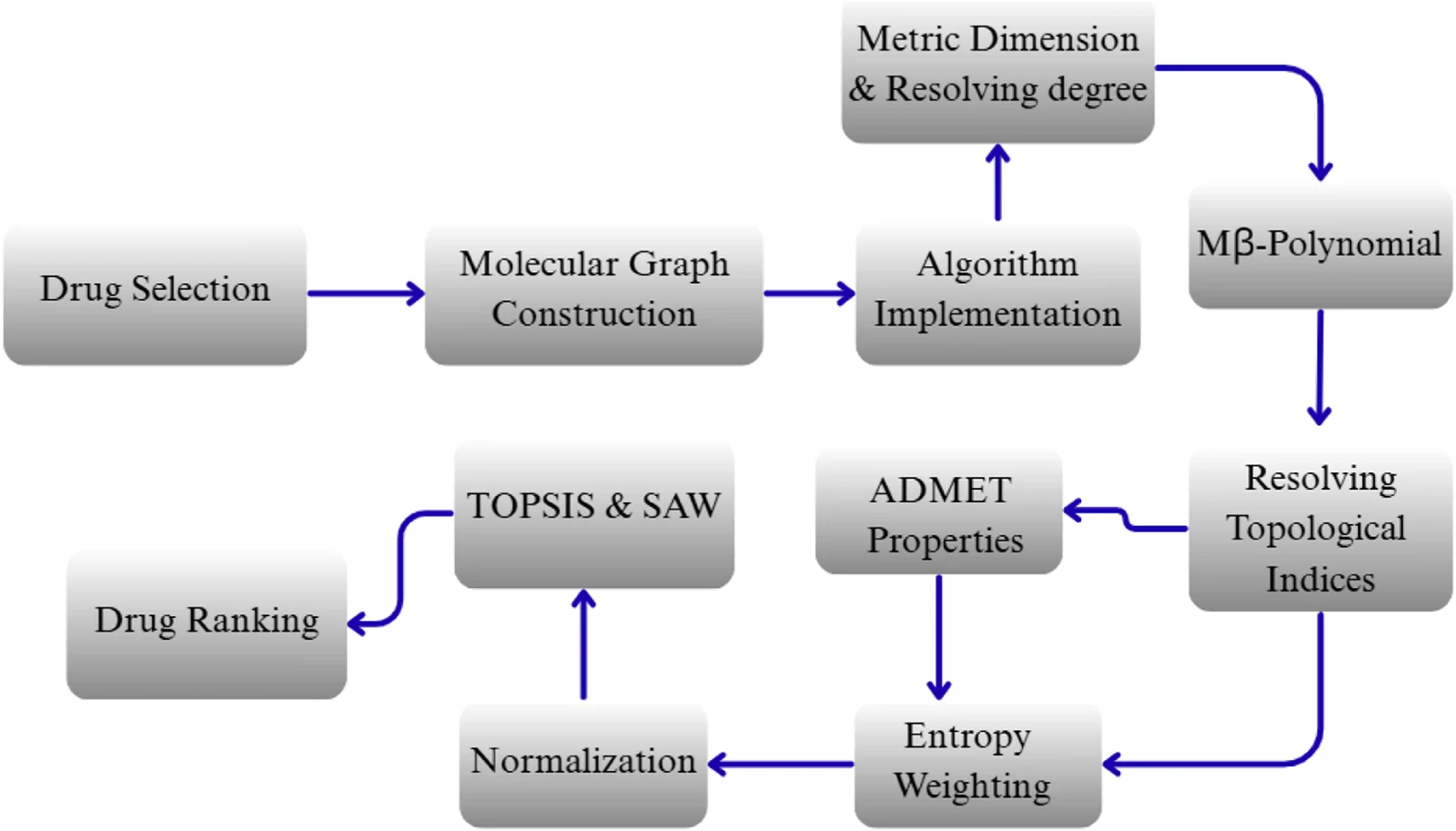
Workflow of the proposed methodology.

## 2 Basic definition

Definition 1 (Chandrakala et al., 2024) *For every point v in V(G), the resolving degree of v is equal to the minimum cardinality of a resolving set of G that contains v, represented by* $d_{\beta_{u}}$ *. The minimum and maximum resolving degrees of G are* $\delta_{\beta }\left(G\right)=\delta_{\beta }=\min\left\{d_{\beta_{v}}:v\in V\left(G\right)\right\}$ *and* $\Delta_{\beta }\left(G\right)=\Delta_{\beta }=\max\left\{d_{\beta_{v}}:v\in V\left(G\right)\right\}$ *, respectively.*

The resolving topological indices (RTIs) are defined as follows:

$${\;\;}_{\beta }M_{1}\left(G\right)=\underset{v\in V\left(G\right)}{\sum }d_{\beta_{v}}^{2},$$

$${\;\;}_{\beta }M_{1}^{*}\left(G\right)=\underset{uv\in E\left(G\right)}{\sum }\left[d_{\beta_{u}}+d_{\beta_{v}}\right],$$

$${\;\;}_{\beta }M_{2}\left(G\right)=\underset{uv\in E\left(G\right)}{\sum }\left[d_{\beta_{u}}\cdot d_{\beta_{v}}\right],$$

$${\;\;}_{\beta }HM\left(G\right)=\underset{uv\in E\left(G\right)}{\sum }{\left[d_{\beta_{u}}+d_{\beta_{v}}\right]}^{2},$$

$${\;\;}_{\beta }F\left(G\right)=\underset{uv\in E\left(G\right)}{\sum }\left[d_{\beta_{u}}^{2}+d_{\beta_{v}}^{2}\right].$$

Several algebraic polynomials were introduced in the literature to compute topological indices from a single polynomial rather than by computing from its definition as is customary. Zagreb (Kamran Siddiqui et al., 2016), Hosoya (Hosoya, 1988), Schultz (Reza Farahani, 2014), Forgotten (Ajmal et al., 2017), Matching and Tutte (Merino et al., 2012), M (Deutsch and Klavžar, 2014), NM (Mondal et al., 2021), RNM (Saleh et al., 2021), and other polynomials are a few examples of significant polynomials. M-polynomials have been used in numerous studies recently to recover a wide range of degree-based topological indices.

Here, the authors introduced and generated the resolving topological indices (Chandrakala et al., 2024) using polynomial methods. The resolving topological indices (RTIs) of the molecules are determined in this study using $M_{\beta }$ -polynomials.

The $M_{\beta }$ -polynomial is stated as $M_{\beta }\left(G;a,b\right)=\sum_{\delta_{\beta }\leq i\leq j\leq \Delta_{\beta }}d_{\beta }m_{\left(i,j\right)}a^{i}b^{j}$ ,

where $d_{\beta }m_{\left(i,j\right)}\left(G\right)=|\left\{e=uv:d_{\beta_{u}}=i,d_{\beta_{v}}=j\right\}|$ . Using Table 2, we can write the resolving indices on E(G) as follows: $I_{\beta }\left(G\right)=\sum_{uv\in E\left(G\right)}f\left(d_{\beta_{u}},d_{\beta_{v}}\right)$ , and the resolving indices from the $M_{\beta }$ -polynomial are shown in Table 3. Here, $D_{a}\left(f\left(a,b\right)\right)=a\frac{\partial \left(f\left(a,b\right)\right)}{\partial a}$ and $D_{b}\left(f\left(a,b\right)\right)=b\frac{\partial \left(f\left(a,b\right)\right)}{\partial b}$.

**TABLE 2:** Detailing of some resolving topological indices.

Resolving index	$f\left(d_{\beta_{u}},d_{\beta_{v}}\right)$
M1*β(G)	$d_{\beta_{u}}+d_{\beta_{v}}$
M2β(G)	$d_{\beta_{u}}\cdot d_{\beta_{v}}$
HMβ(G)	${\left[d_{\beta_{u}}+d_{\beta_{v}}\right]}^{2}$
Fβ(G)	$d_{\beta_{u}}^{2}+d_{\beta_{v}}^{2}$

**TABLE 3:** Resolving indices and their derivation from $M_{\beta }$ -polynomials.

Resolving index	Derivation from
M1*β(G)	$\left(D_{a}+D_{b}=\right)\cdot M_{\beta }\left(G\right){|}_{a=b=1}$
M2β(G)	$\left(D_{a}\cdot D_{b}\right)\cdot M_{\beta }\left(G\right){|}_{a=b=1}$
HMβ(G)	${\left(D_{a}+D_{b}\right)}^{2}\cdot M_{\beta }\left(G\right){|}_{a=b=1}$
Fβ(G)	$\left({D_{a}}^{2}+{D_{b}}^{2}\right)\cdot M_{\beta }\left(G\right){|}_{a=b=1}$

This article shows how to get the resolving topological indices of (G) for several breast cancer drugs using the $M_{\beta }$ -polynomial.

Algorithm 1 Brute-force algorithm for finding minimal resolving sets and the minimum size.Note: The brute-force algorithm exhibits high computational complexity. Therefore, it is suitable mainly for small to moderately sized structures.

Input: A connected undirected graph G=(V,E) .
Output: All minimal resolving sets and their size.
FUNCTION is_resolving_set(graph, node_set)
Let V← list of all vertices in G .
for r from 1 to |V| , do:
  for each subset S⊆V such that |S|=r , do:
   Initialize distance_vectors ← empty map.
   Set is_resolving ←True for each vertex u∈V , do:
    Let D(u)← empty list.
    for each vertex s∈S , do:
      Compute d←shortest_path_length(G,u,s) .
      Append d to D(u)

    if D(u) already exists in distance_vectors, then:
      Set is_resolving ←False .
      break.
    else:
      Add mapping D(u)→u to distance_vectors.
   if is_resolving = True, then:
    return S .
 return None
 FUNCTION find_all_minimal_resolving_sets(graph)
  Let nodes be the list of nodes in a graph.
  Let n be the number of nodes.
  Initialize minimal_sets as an empty list.
  Set min_size to n.
  for r from 1 to n :
   if r>min_size and minimal_sets is not empty, then:
    break.
   for every candidate in every set of node combinations of size r :
    if is_resolving_set(graph, candidate) is True:
     if r is lessthan min_size:
      minimal_sets ←[candidate]

       min_size ←r

     else if r=min_size :
      Add candidate to minimal_sets.
  return (min_size, minimal_sets).
 Execution
 Call the procedure to find_all_minimal_resolving_sets(G) and min_size(G)

## 3 Main results and discussions

### 3.1 Computation of $M_{\beta }$ -polynomial using resolving topological indices

This section calculates the $M_{\beta }$ -polynomial of a given molecular graph using the resolving topological indices. These indices, which reflect the resolving characteristics of vertices in the graph, give a more detailed structural representation for computing the $M_{\beta }$ -polynomial. By methodically adding the resolving parameters, the resultant $M_{\beta }$ -polynomial captures both the adjacency and differentiating features of the graph. The exact expressions and resulting coefficients are shown below to illustrate the computation process. In the following theorem, we calculate the $M_{\beta }$ -polynomial of the molecular graph of tamoxifen.

Theorem 1 *If* G *is a simple connected graph of tamoxifen, then*

1. *the* $M_{\beta }$ *-polynomial of tamoxifen is*

$$M_{\beta }\left(G;a,b\right)=10a^{5}b^{5}+14a^{4}b^{5}+6a^{4}b^{4}.$$

2. M1*β(G)=274,M2β(G)=626,HMβ(G)=2518,Fβ(G)=1266,M1β(G)=574 *.*

Proof:

1. Let G be the molecular graph of tamoxifen, as illustrated in Figure 3, with |V|=28 and |E|=30 . Using the edge partition of tamoxifen molecular graph (Table 4) and the concept of $M_{\beta }$ -polynomial, we obtain the required expression. Let $d_{\beta }m_{\left(i,j\right)}\left(G\right)=|\left\{e=uv:d_{\beta_{u}}=i,d_{\beta_{v}}=j\right\}|$ .

$$\begin{array}{ll} M_{\beta }\left(G;a,b\right) & =\underset{\delta_{\beta }\leq i\leq j\leq \Delta_{\beta }}{\sum }d_{\beta }\;m_{\left(i,j\right)}\;a^{i}b^{j}\\ & =10a^{5}b^{5}+14a^{4}b^{5}+6a^{4}b^{4}. \end{array}$$

2. With the help of its $M_{\beta }$ -polynomial and derivation formula, which are compiled in Table 3, the resolving-degree-based indices of the molecular graph of tamoxifen are examined.

$$\begin{array}{l} D_{a}\left[M_{\beta }\left(G;a,b\right)\right]=a\left(50a^{4}b^{5}+56a^{3}b^{5}+24a^{3}b^{4}\right)\\ D_{a}^{2}\left[M_{\beta }\left(G;a,b\right)\right]=a\left(50a^{4}b^{5}+56a^{3}b^{5}+24a^{3}b^{4}+a\left(200a^{3}b^{5}+168a^{2}b^{5}+72a^{2}b^{4}\right)\right)\\ D_{b}\left[M_{\beta }\left(G;a,b\right)\right]=b\left(50a^{5}b^{4}+70a^{4}b^{4}+24a^{4}b^{3}\right)\\ D_{b}^{2}\left[M_{\beta }\left(G;a,b\right)\right]=b\left(50a^{5}b^{4}+70a^{4}b^{4}+24a^{4}b^{3}+b\left(200a^{5}b^{3}+280a^{4}b^{3}+72a^{4}b^{2}\right)\right)\\ \left(D_{a}+D_{b}\right)\left[M_{\beta }\left(G;a,b\right)\right]=a\left(50a^{4}b^{5}+56a^{3}b^{5}+24a^{3}b^{4}\right)+b\left(50a^{5}b^{4}+70a^{4}b^{4}+24a^{4}b^{3}\right)\\ \left(D_{a}D_{b}\right)\left[M_{\beta }\left(G;a,b\right)\right]=ab\left(250a^{4}b^{4}+280a^{3}b^{4}+96a^{3}b^{3}\right)\\ {\left(D_{a}+D_{b}\right)}^{2}\left[M_{\beta }\left(G;a,b\right)\right]=2ab\left(250a^{4}b^{4}+280a^{3}b^{4}+96a^{3}b^{3}\right)\\ \;+a\left(50a^{4}b^{5}+56a^{3}b^{5}+24a^{3}b^{4}+200a^{4}b^{5}+168a^{3}b^{5}+72a^{3}b^{4}\right)\\ \;+b\left(50a^{5}b^{4}+70a^{4}b^{4}+24a^{4}b^{3}+200a^{5}b^{4}+280a^{4}b^{4}+72a^{4}b^{3}\right)\\ \left(D_{a}^{2}+D_{b}^{2}\right)\left[M_{\beta }\left(G;a,b\right)\right]=a\left(50a^{4}b^{5}+56a^{3}b^{5}+24a^{3}b^{4}+a\left(200a^{3}b^{5}+168a^{2}b^{5}+72a^{2}b^{4}\right)\right)\\ \;+b\left(50a^{5}b^{4}+70a^{4}b^{4}+24a^{4}b^{3}+b\left(200a^{5}b^{3}+280a^{4}b^{3}+72a^{4}b^{2}\right)\right). \end{array}$$

Therefore,

$$\begin{array}{ll} {\;\;}_{\beta }M_{1}^{*}\left(G\right) & =\left(D_{a}+D_{b}\right)\cdot M_{\beta }\left(G\right){|}_{a=b=1}=274\\ {\;\;}_{\beta }M_{2}\left(G\right) & =\left(D_{a}\cdot D_{b}\right)\cdot M_{\beta }\left(G\right){|}_{a=b=1}=626\\ {\;\;}_{\beta }HM\left(G\right) & ={\left(D_{a}+D_{b}\right)}^{2}\cdot M_{\beta }\left(G\right){|}_{a=b=1}=2518\\ {\;\;}_{\beta }F\left(G\right) & =\left({D_{a}}^{2}+{D_{b}}^{2}\right)\cdot M_{\beta }\left(G\right){|}_{a=b=1}=1266\\ {\;\;}_{\beta }M_{1}\left(G\right) & =\underset{v\in V\left(G\right)}{\sum }d_{\beta_{v}}^{2}=574. \end{array}$$

**FIGURE 3:**
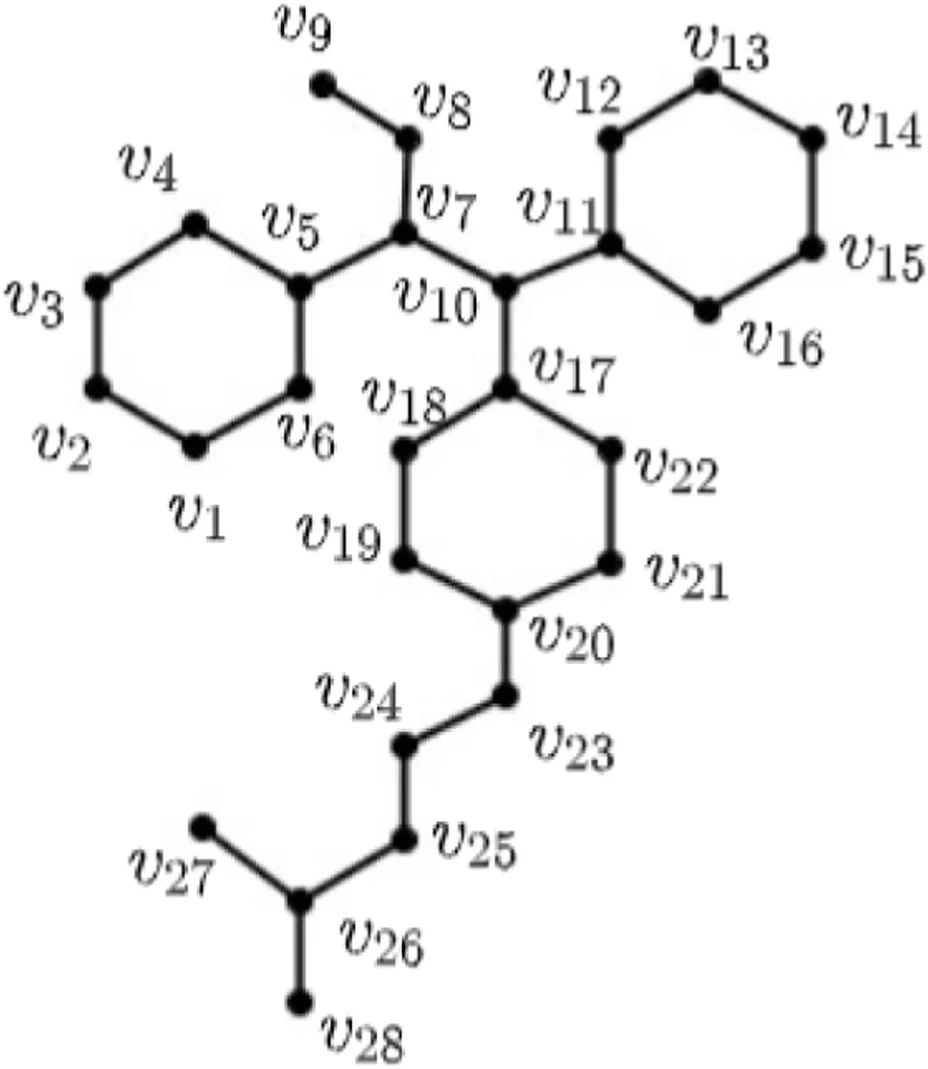
Molecular graph of tamoxifen.

**TABLE 4:** Edge partition of tamoxifen and $d_{\beta_{v}}$ of each vertices of tamoxifen.

$\left(d_{\beta_{u}},d_{\beta_{v}}\right)$	(5,5)	(4,5)	(4,4)
Number of edges	10	14	6
$d_{\beta_{v}}$	4	5	
Number of vertices	14	14	

This completes the proof.

As shown in Figure 4, the 3d surface plot of the $M_{\beta }$ -polynomial derived from the tamoxifen structure illustrates a non-linear growth in locations with higher a and b values. This indicates the molecules’ sensitivity to descriptor modifications and offers information about its topological complexity for QSPR studies. These results are obtained using the same methodology as described in Theorem 1 (Ahmed et al., 2026), based on the metric dimensions, edge partition, and resolving degrees of the corresponding molecular graphs, and are summarized in Table 5.

**FIGURE 4:**
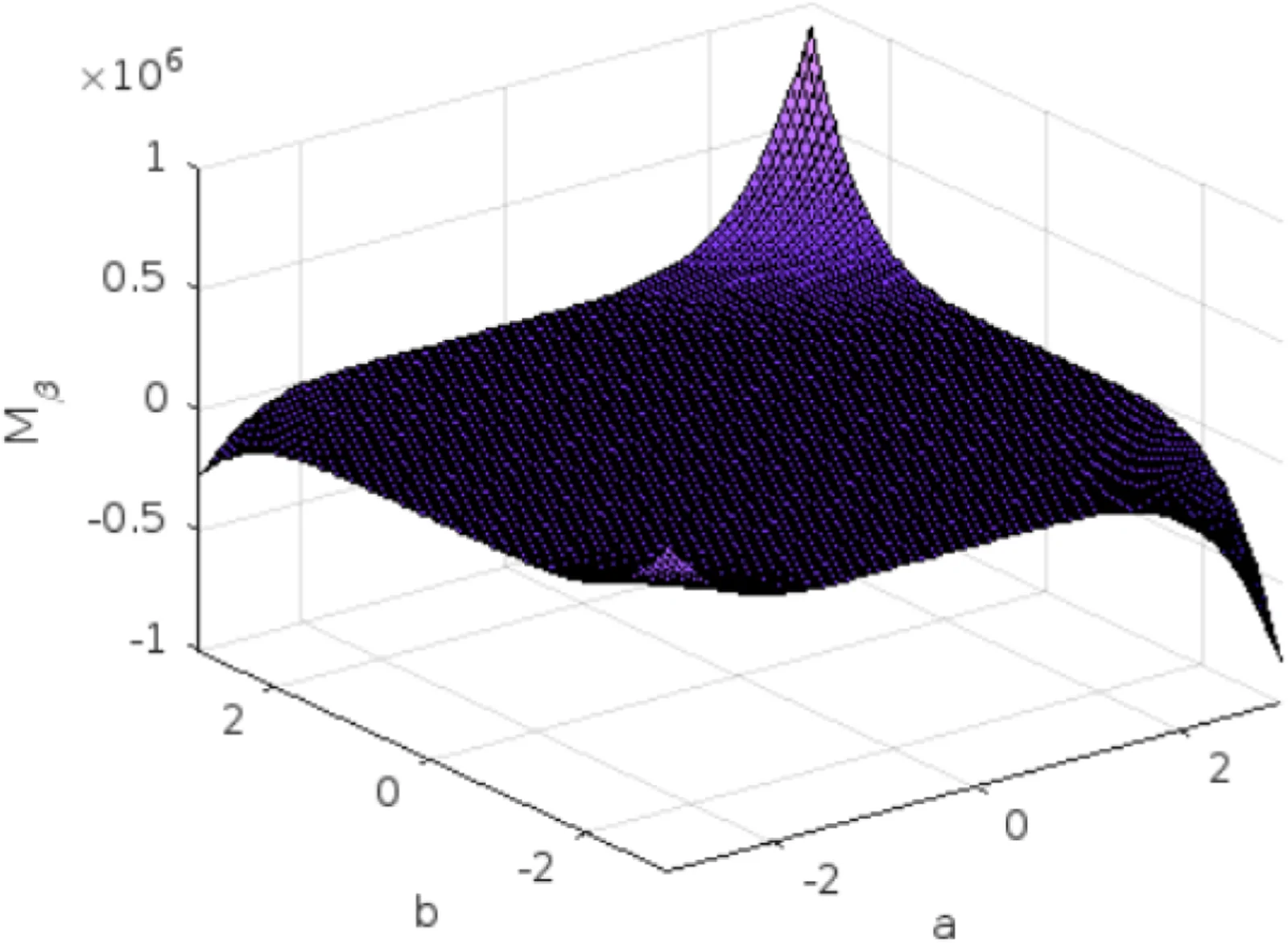
Three-dimensional surface plot of M_β_ for Tamoxifen with respect to a and b.

**TABLE 5:** Calculations of the $M_{\beta }$ -polynomial expressions and metric dimension β(G) for the molecular graphs of the chosen breast cancer drugs.

Drugs	β(G)	$M_{\beta }$ -polynomial
Tamoxifen	4	$10a^{5}b^{5}+14a^{4}b^{5}+6a^{4}b^{4}$
Elacestrant	2	$25a^{3}b^{3}+8a^{2}b^{3}+4a^{2}b^{2}$
Toremifene	4	$11a^{5}b^{5}+14a^{5}b^{4}+6a^{4}b^{4}$
Fulvestrant	5	$20a^{6}b^{6}+11a^{5}b^{6}+13a^{5}b^{5}$
Megestrol acetate	4	$17a^{5}b^{5}+9a^{5}b^{4}+5a^{4}b^{4}$
Anastrozole	5	$10a^{6}b^{6}+8a^{5}b^{6}+5a^{5}b^{5}$
Exemestane	3	$7a^{4}b^{4}+7a^{3}b^{4}+11a^{3}b^{3}$
Letrozole	3	$7a^{4}b^{4}+10a^{3}b^{4}+7a^{3}b^{3}$
Alpelisib	4	$25a^{5}b^{5}+7a^{4}b^{5}$
Ixabepilone	3	$16a^{4}b^{4}+8a^{3}b^{4}+13a^{3}b^{3}$
Abemaciclib	3	$7a^{4}b^{4}+10a^{4}b^{3}+4a^{3}b^{3}$
Capivasertib	3	$15a^{4}b^{4}+12a^{3}b^{4}+6a^{3}b^{3}$
Ribociclib	4	$17a^{5}b^{5}+12a^{4}b^{5}+7a^{4}b^{4}$
Neratinib	4	$16a^{5}b^{5}+14a^{4}b^{5}+13a^{4}b^{4}$
Olaparib	3	$23a^{4}b^{4}+10a^{3}b^{4}+3a^{3}b^{3}$

The resolving topological indices were computed using the metric dimensions and $M_{\beta }$ -polynomials that were extracted from the smallest resolving sets using Algorithm 1.

Figure 5 shows the 3D plot of $M_{\beta }\left(G;a,b\right)$ for elacestrant that depicts how resolving indices vary with different values of a and b. This representation aids in understanding the structural complexity and topological aspects embedded in the polynomial.

**FIGURE 5:**
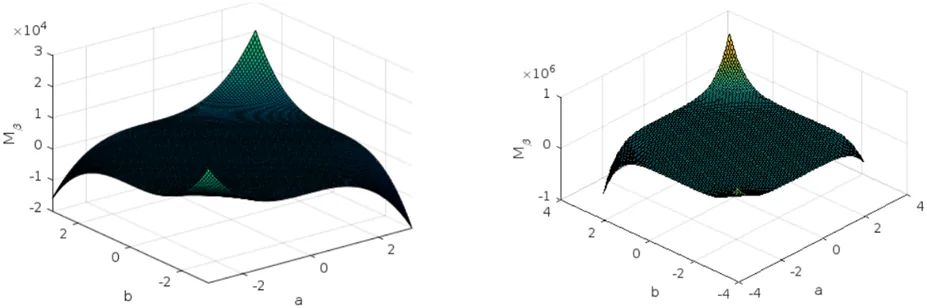
Three-dimensional surface plot of M_β_ for elacestrant and toremifene with respect to a and b.

As shown in Figure 5, the 3D plot clearly shows how the resolving indices respond to various parameter values, which indicate the drug’s molecular complexity. Similarly, Figures 6–10 illustrate the 3D surface plots of the resolving index polynomials for all the studied drugs, including fulvestrant, megestrol acetate, anastrozole, exemestane, letrozole, alpelisib, ixabepilone, abemaciclib, capivasertib, ribociclib, neratinib, and olaparib. The plots demonstrate how topological indices alter with varying a and b values, reflecting the distinct structural properties of each molecule. The non-linear patterns observed indicate that the indices are sensitive to changes in molecular connectivity and branching. This behavior illustrates the influence of structural complexity on physicochemical and pharmacokinetic properties (e.g., solubility and bioavailability) in the QSPR framework.

**FIGURE 6:**
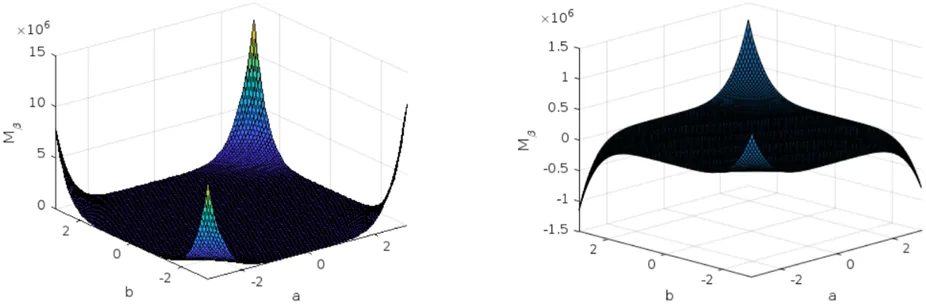
Three-dimensional surface plot of M_β_ for fulvestrant and megestrol acetate with respect to a and b.

**FIGURE 7:**
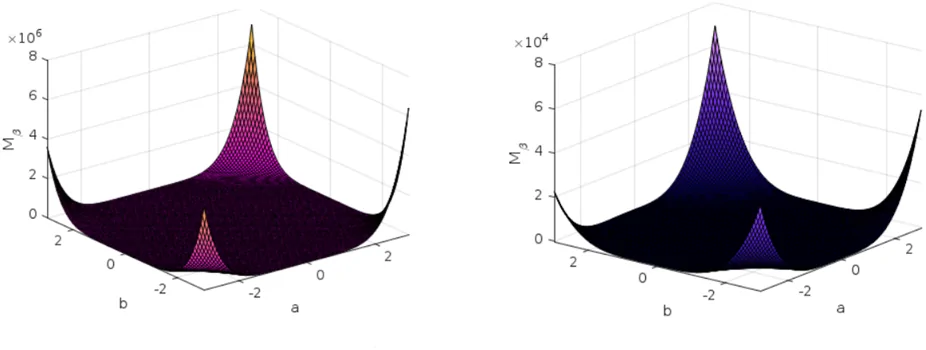
Three-dimensional surface plot of M_β_ for anastrozole and exemestane with respect to a and b.

**FIGURE 8:**
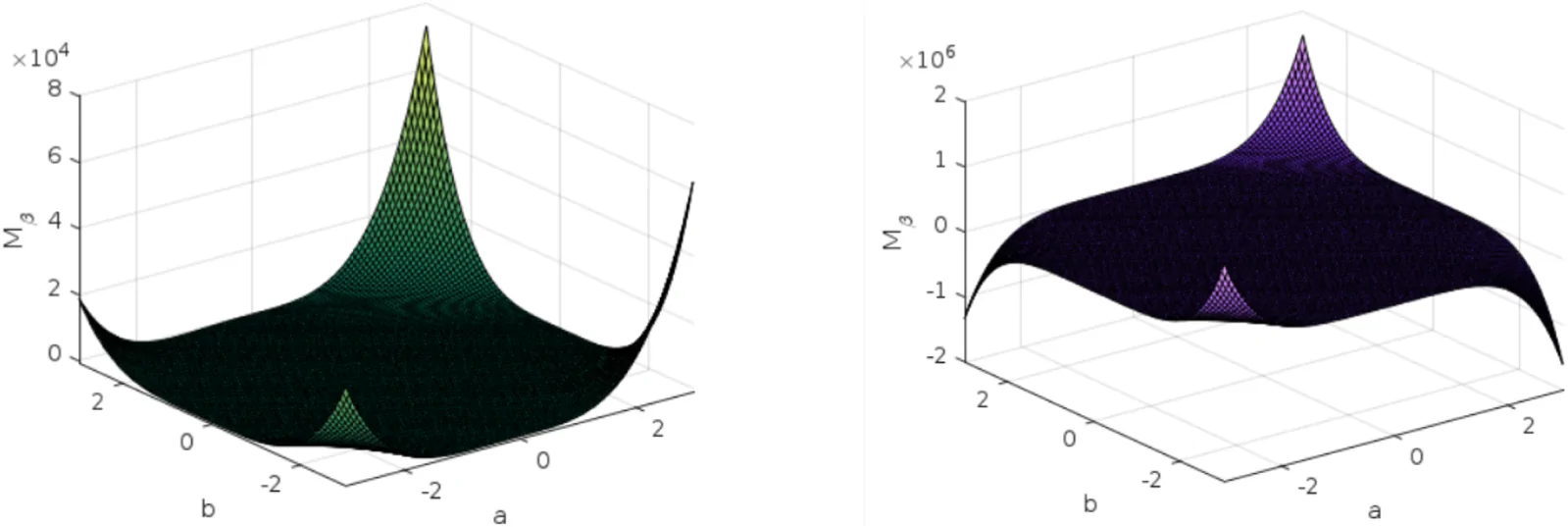
Three-dimensional surface plot of M_β_ for letrozole and alpelisib with respect to a and b.

**FIGURE 9:**
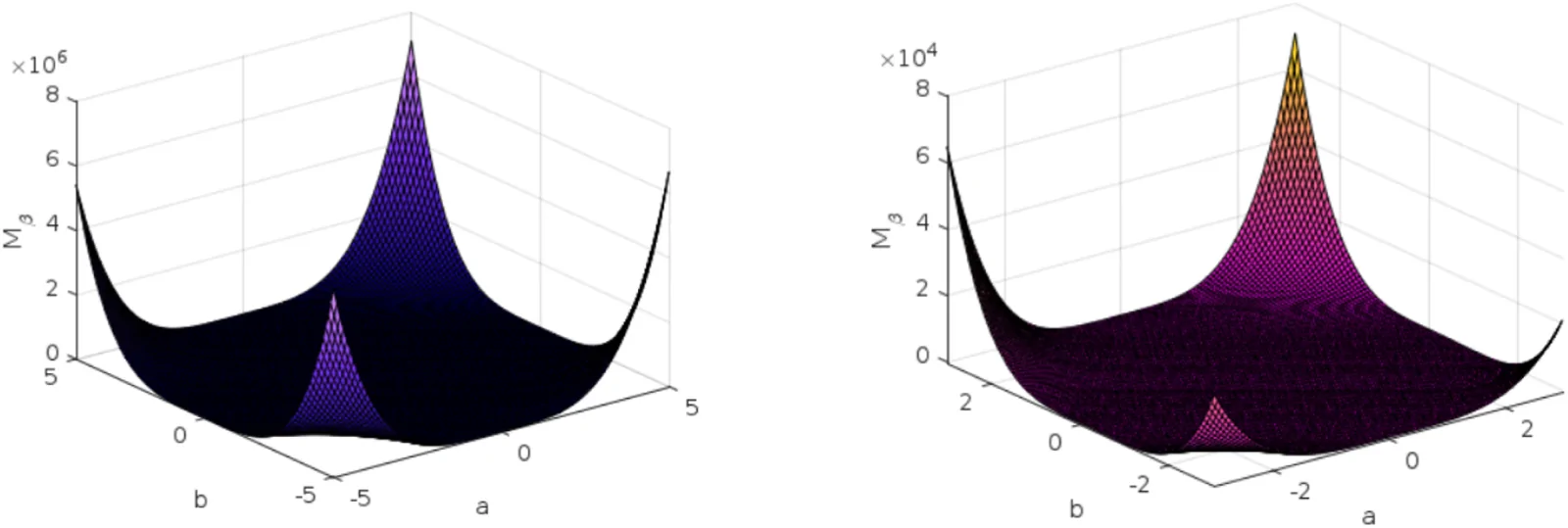
Three-dimensional surface plot of M_β_ for ixabepilone and abemaciclib with respect to a and b.

**FIGURE 10:**
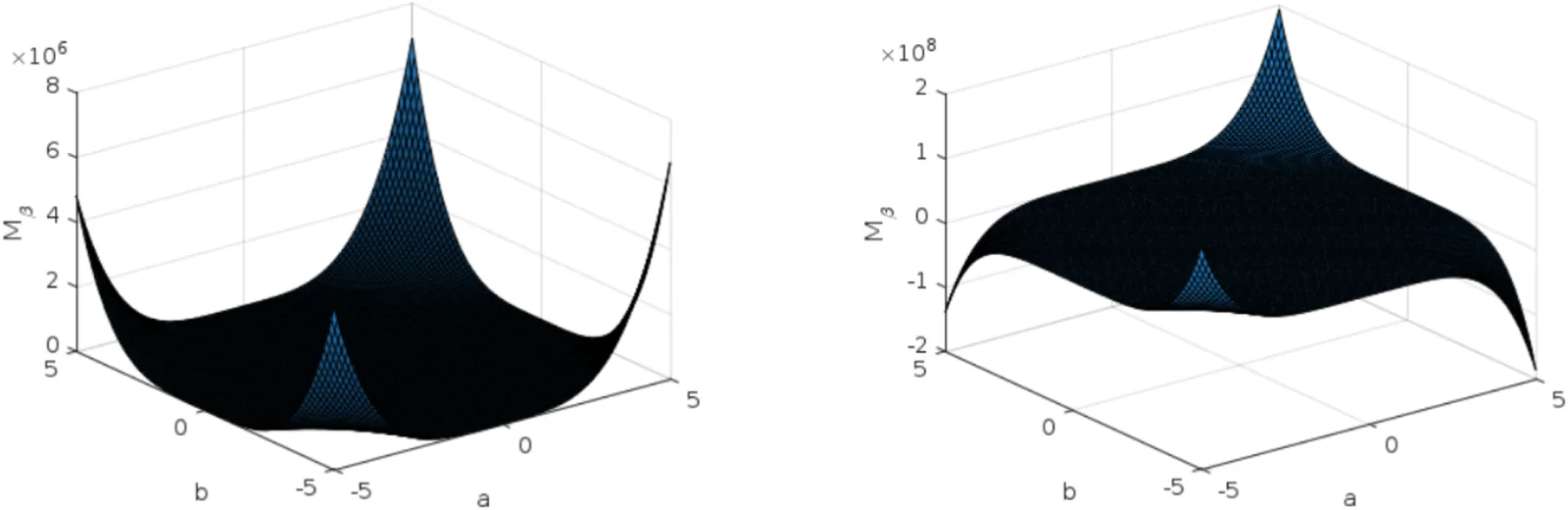
Three-dimensional surface plot of M_β_ for capivasertib and ribociclib with respect to a and b.

**FIGURE 11:**
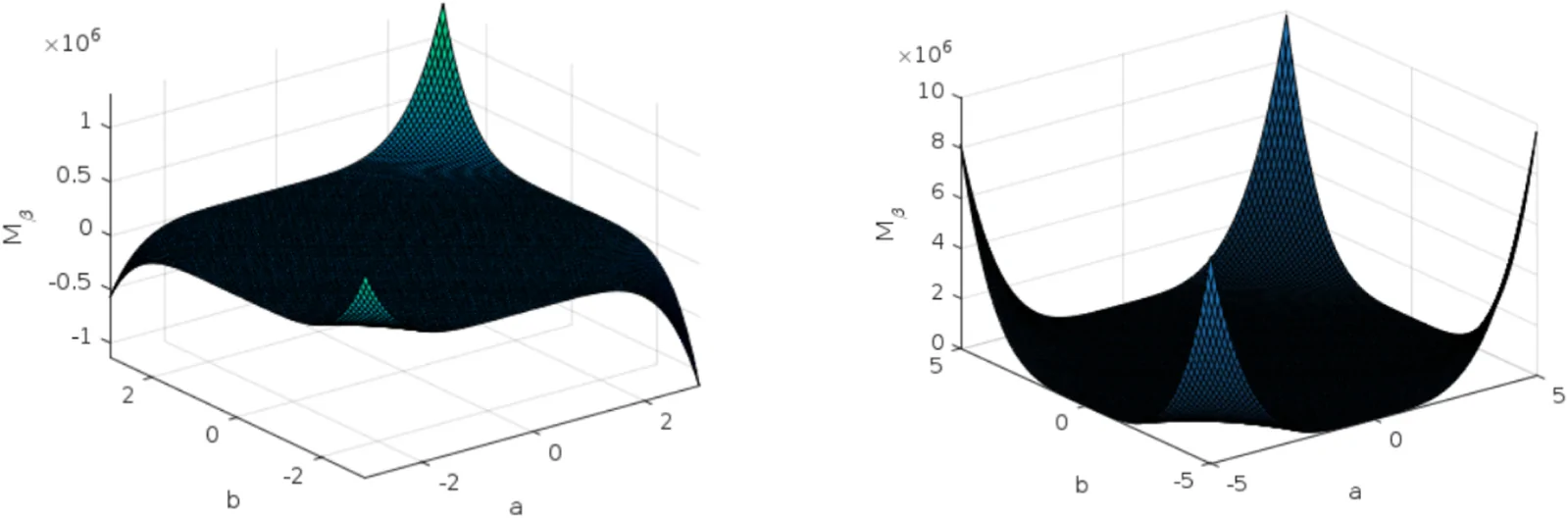
Three-dimensional surface plot of M_β_ for neratinib and olaparib with respect to a and b.

### 3.2 Regression analysis

#### 3.2.1 Quadratic regression

Quadratic regression is a statistical tool for analyzing relationships between a dependent variable and one or more independent variables. Quadratic regression describes curved relationships by fitting a second-order polynomial, which helps in prediction accuracy and understanding complex datasets. The model is described by the following equation:

$$\hat{y}={\hat{\lambda }}_{0}+{\hat{\lambda }}_{1}x+{\hat{\lambda }}_{2}x^{2}.$$

In the quadratic regression model, $\hat{y}$ is the dependent variable, x is the independent variable, ${\hat{\lambda }}_{0}$ is the constant (intercept), and ${\hat{\lambda }}_{1}$ and ${\hat{\lambda }}_{2}$ are the regression coefficients assigned to quadratic terms, respectively.

$$ORCT=2.0863-0.001485{\;\;}_{\beta }M_{1}\left(G\right)+3.5441\times 10^{-6}\;{\left({\;\;}_{\beta }M_{1}\left(G\right)-555.467\right)}^{2},$$

$$ORCT=2.1738-0.0032129{\;\;}_{\beta }M_{1}^{*}\left(G\right)+3.3535\times 10^{-5}\;{\left({\;\;}_{\beta }M_{1}^{*}\left(G\right)-284.6\right)}^{2},$$

$$ORCT=1.9804-0.0011622{\;\;}_{\beta }M_{2}\left(G\right)+2.958\times 10^{-6}\;{\left({\;\;}_{\beta }M_{2}\left(G\right)-626.8\right)}^{2},$$

$$ORCT=1.9819-0.00029{\;\;}_{\beta }HM\left(G\right)+1.8479\times 10^{-7}\;{\left({\;\;}_{\beta }HM\left(G\right)-2517.4\right)}^{2},$$

$$ORCT=1.9833-0.0005788{\;\;}_{\beta }F\left(G\right)+7.3881\times 10^{-7}\;{\left({\;\;}_{\beta }F\left(G\right)-1263.8\right)}^{2}.$$

A weak-to-moderate correlation between the calculated topological indices and the chosen ADMET features is demonstrated using the quadratic regression analysis. ORCT has a moderate correlation with all indices, with R values ranging from 0.618 to 0.67, indicating a moderate predictive capability. Similarly, ORAT shows a weak-to-moderate correlation, notably with M1*β(G) (R=0.578) , suggesting that it is responsive to structural variations.

In contrast, WS, Caco-2, and SP exhibit poor correlations, with R values usually lower than 0.55, suggesting poor-to-weak dependency on the investigated topological indices. Overall, the indices M2β(G) , HMβ(G) , and Fβ(G) have better and more consistent correlations across most ADMET parameters than M1β(G) and M1*β(G) . To validate the QSPR relationship, correlation coefficients were calculated between the resolving topological indices and each ADMET property (WS, Caco-2, SP, ORAT, and ORCT). The results are presented in Table 6.

**TABLE 6:** Correlation coefficients (R) for quadratic regression.

Property	M1β(G)	M1*β(G)	M2β(G)	HMβ(G)	Fβ(G)
WS	0.517	0.482	0.523	0.523	0.524
Caco-2	0.372	0.395	0.368	0.417	0.369
SP	0.307	0.366	0.323	0.323	0.323
ORAT	0.470	0.578	0.467	0.467	0.467
ORCT	0.670	0.618	0.656	0.656	0.656

The results indicate that quadratic regression models have a moderate association between ORCT and resolving topological indices. In Table 7, M1β(G) outperforms all other indices with the highest $R^{2}$ value (0.4495) and lowest error. The indices M2β(G) , HMβ(G) , and Fβ(G) yield comparable findings with $R^{2}$ values around 0.43, indicating the models’ stability. The index M1*β(G) produces significantly weaker findings, with a lower $R^{2}$ value (0.3817). However, all models are statistically significant, with p-values less than 0.05. The models exhibit a moderate non-linear connection between ORCT and the topological indices, and they are generally acceptable. Overall, predictions are generally poor for most properties, except ORCT, which has moderate accuracy. Figure 12 demonstrates the comparison between observed ORCT and anticipated values using the resolving topological indices M1β(G) , M1*β(G) , M2β(G) , HMβ(G) , and Fβ(G) . The findings demonstrate that M2β(G) , HMβ(G) , and Fβ(G) exhibit comparable patterns, whereas M1*β(G) and M1*β(G) show little differences.

**TABLE 7:** Statistical parameters of quadratic regression models for ORCT.

RTI	$R^{2}$	Adj $R^{2}$	Se	F	*p*-value
M1β(G)	0.4495	0.3625	0.4270	4.9002	0.03
M1*β(G)	0.3817	0.2737	0.4526	3.7036	0.05
M2β(G)	0.4299	0.3326	0.4345	4.5256	0.03
HMβ(G)	0.4301	0.3326	0.4345	4.5270	0.03
Fβ(G)	0.4302	0.3327	0.4345	4.5290	0.03

**FIGURE 12:**
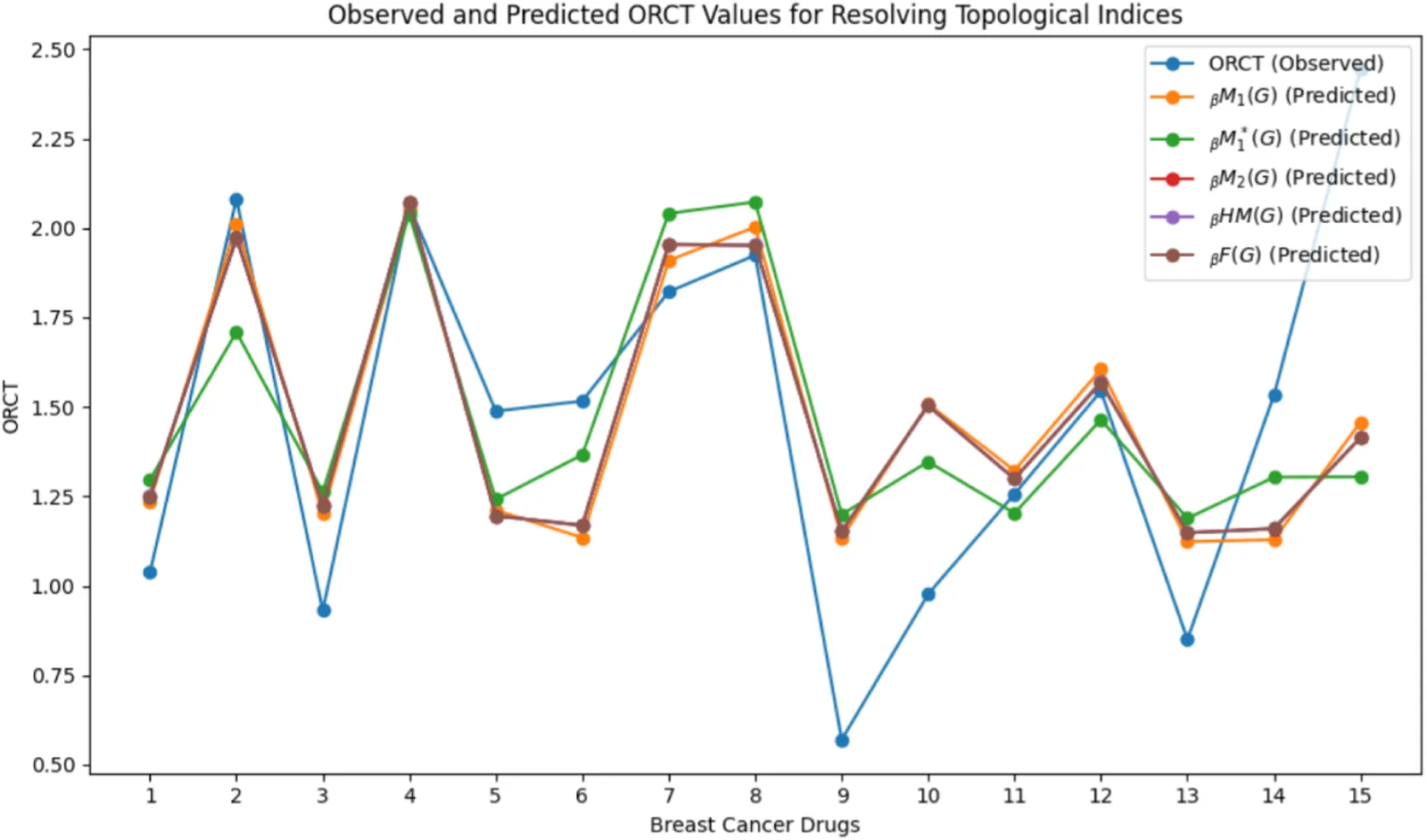
Comparison of the observed ORCT values with the expected values calculated using the resolving topological indices.

### 3.3 Combining QSPR insights and MCDM techniques for rational selection

In this study, it is essential to consider the QSPR link between these resolving indices (Table 8) and the ADMET properties (Table 9) of the breast cancer drugs being studied, including WS, Caco-2, SP, ORAT, and ORCT. The ADMET properties water solubility (WS), Caco-2 permeability, skin permeability (SP), oral acute toxicity (ORAT), and oral chronic toxicity were selected for this study because they demonstrate essential drug characteristics, including the drug’s ability to dissolve, absorb, and cross membranes, along with its short-term and long-term safety. (i) WS shows how well the drug dissolves in water, improving oral absorption and bioavailability. (ii) Better intestinal absorption across the gut epithelium is indicated by a greater Caco-2 permeability (Caco-2) score. (iii) Skin permeability (SP) indicates the drug’s ability to be delivered through the skin; higher skin penetration is indicated by lower logKp (more negative) values. (iv) A safer profile and less acute toxicity are indicated by a greater oral acute toxicity (ORAT) value. (v) A medicine with a higher oral chronic toxicity (ORCT) rating is less toxic after prolonged exposure, which guarantees improved safety during long-term therapy. These combined attributes serve as a reference for multicriteria ranking that takes safety and efficacy into consideration.

**TABLE 8:** Computed Resolving Topological Indices.

Drugs	M1β(G)	M1*β(G)	M2β(G)	HMβ(G)	Fβ(G)
Tamoxifen	574	274	626	2,518	1,266
Elacestrant	266	206	289	1,164	586
Toremifene	599	284	651	2,618	1,316
Fulvestrant	1,328	491	1,375	5,511	2,761
Megestrol acetate	592	291	685	2,749	1,379
Anastrozole	660	258	725	2,908	1,458
Exemestane	295	171	295	1,187	597
Letrozole	268	168	295	1,190	600
Alpelisib	662	313	765	3,067	1,537
Ixabepilone	434	262	469	1,884	946
Abemaciclib	522	310	588	2,362	1,186
Capivasertib	396	240	438	1,764	888
Ribociclib	674	334	777	3,120	1,566
Neratinib	667	390	888	3,566	1790
Olaparib	456	272	515	2,070	1,040

**TABLE 9:** ADMET properties for breast cancer drugs.

Drug	WS	Caco-2	SP	ORAT	ORCT
Tamoxifen	−5.891	1.312	−2.664	2.349	1.037
Elacestrant	−5.668	1.268	−2.833	2.45	2.081
Toremifene	−6.154	1.315	−2.704	2.331	0.931
Fulvestrant	−6.9	1.293	−2.765	2.591	2.063
Megestrol acetate	−5.483	1.218	−3.531	2.259	1.488
Anastrozole	−4.346	1.053	−2.973	2.485	1.516
Exemestane	−5.176	1.523	−2.891	2.062	1.822
Letrozole	−4.226	1.128	−2.961	2.4	1.924
Alpelisib	−5.493	0.236	−3.256	3.315	0.57
Ixabepilone	−5.472	1.119	−3.182	2.591	0.976
Abemaciclib	−3.158	0.853	−2.777	3.038	1.256
Capivasertib	−3.653	0.169	−3.276	2.783	1.544
Ribociclib	−3.882	1.04	−3.323	3.244	0.85
Neratinib	−5.509	0.415	−2.835	2.749	1.532
Olaparib	−4.374	1.187	−3.33	2.605	2.443

TOPSIS and SAW were chosen because they are simple, efficient, and well-known MCDM approaches for ranking options. They are computationally simpler than approaches such as AHP and VIKOR, which is appropriate for the present study. Before we explore the field of MCDM, we should review the fundamentals. In our situation, we use anticancer drugs as our alternatives and resolving topological indices (RTIs) as our criteria. Both beneficiary and non-beneficiary factors are taken into consideration when using MCDM approaches to assess alternatives. Higher values are chosen for positive influence in beneficiary criteria, whereas lower values are preferable for non-beneficiary criteria. Several methods were used in this study to assess the importance of index weights when allocating weights to ADMET features. The entropy approach was one of the techniques employed.

### 3.4 Entropy method for weight allocation

The entropy method is an objective weighing approach that determines the significance of each criterion based on data variability. It computes the entropy for each criterion, which indicates the level of disorder or uncertainty. Criteria with more variability receive higher weights because they give more information for decision-making. The entropy approach is used to provide objective, data-driven weight allocation while avoiding subjective bias. This method has been widely employed in recent QSPR and drug ranking analysis, where variability-based weighting improves the reliability and transparency of the decision-making process. We used the entropy method to figure out the objective weights for each criterion. The first step was to make the decision matrix and use Equation 1 to normalize it so that the raw data could be compared. Equation 2 was then used to find the entropy value for each criterion. This equation quantifies the degree of disorder or uncertainty. Using this information, we were able to find the degree of diversification using Equation 3, and then we used Equation 4 to find the weights. Table 12 shows the calculated weights, which makes sure that the weighting process is based only on data and not on personal opinion.

$$N_{ij}=\frac{Z_{ij}}{\sum_{i=1}^{m}Z_{ij}},1\leq i,j\leq m,n,$$ (1)

$$E_{j}=-l\overset{m}{\underset{i=1}{\sum }}N_{ij}\ln\left(N_{ij}\right),\;\text{where}\;l=\frac{1}{\ln\left(m\right)}.$$ (2)

**TABLE 12:** Values of weight.

$W_{j}^{*}$	0.213409	0.154894	0.210901	0.210563	0.210233

The normalization constant, $l=\frac{1}{\ln\left(m\right)}$ , is based on the maximum Shannon entropy limit and ensures $E_{j}\in \left[0,1\right]$ .

$$D_{j}=|1-E_{j}|,$$ (3)

$$W_{j}^{*}=\frac{D_{j}}{\sum_{j=1}^{n}D_{j}}.$$ (4)

### 3.5 Technique for Order Preference by Similarity to Ideal Solution (TOPSIS) for ranking drugs

TOPSIS is a method for comparing different alternatives based on multiple criteria. This process involves assigning a weight to each criterion, normalizing the scores for comparability, and determining the distance of each option from the ideal solution, which represents the optimal performance for every criterion (Naeem et al., 2021; Roghanian and Kebria, 2017). TOPSIS assumes that the criteria should always go up or down. The TOPSIS framework classifies criteria according to whether they should be minimized or maximized. As a result, while creating the ideal best and ideal worst solutions, WS, Caco-2, ORCT, and ORAT are considered positive (maximization) criteria, while SP is treated as a non-beneficial criterion for minimization. Normalization is very important when there are multiple criteria because the units and scales of the criteria may be different. Normalization was used to transform all values to a single scale and eliminate the effect of varied numerical ranges.

Step 1: Make an evaluation matrix with m options and n criteria. Each criterion and alternative’s intersection should be represented by $z_{ij}$ , and Table 8 contains a matrix similar to ${\left(z_{ij}\right)}_{\left(m\times n\right)}$ .

Step 2: The matrix is then created by standardizing the ${\left(z_{ij}\right)}_{\left(m\times n\right)}$ matrix. $T={\left(t_{ij}\right)}_{\left(m\times n\right)}$ , using the matrix that has been normalized (Table 10).

$$t_{ij}=\frac{z_{ij}}{\sqrt{\sum_{i=1}^{m}z_{ij}^{2}}},\;i=1,2,\ldots ,m\;\&\;j=1,2,\ldots ,n.$$

**TABLE 10:** Normalized decision matrix.

Drugs	M1β(G)	M1*β(G)	M2β(G)	HMβ(G)	Fβ(G)
Tamoxifen	0.241901	0.239769	0.237302	0.237779	0.238252
Elacestrant	0.112100	0.180265	0.109553	0.109918	0.110281
Toremifene	0.252436	0.248520	0.246779	0.247222	0.247662
Fulvestrant	0.559659	0.429660	0.521230	0.520413	0.519600
Megestrol acetate	0.249486	0.254646	0.259667	0.259593	0.259518
Anastrozole	0.278144	0.225768	0.274830	0.274607	0.274385
Exemestane	0.124322	0.149637	0.111828	0.112090	0.112351
Letrozole	0.112943	0.147012	0.111828	0.112374	0.112916
Alpelisib	0.278987	0.273897	0.289993	0.289622	0.289252
Ixabepilone	0.182901	0.229268	0.177787	0.177909	0.178030
Abemaciclib	0.219986	0.271272	0.222897	0.223048	0.223197
Capivasertib	0.166886	0.210017	0.166035	0.166577	0.167115
Ribociclib	0.284044	0.292274	0.294542	0.294627	0.294710
Neratinib	0.281094	0.341277	0.336620	0.336743	0.336865
Olaparib	0.192172	0.238019	0.195224	0.195473	0.195720

Step 3: The weighted normalized decision matrix $X_{ij}$ (Table 11) should be calculated in step 3. The following formula is used to distribute the weight (Table 12):

$$\overset{j}{\underset{i=1}{\sum }}w_{j}^{*}=1.$$

**TABLE 11:** Weighted normalized decision matrix.

Drugs	M1β(G)	M1*β(G)	M2β(G)	HMβ(G)	Fβ(G)
Tamoxifen	0.051624	0.037139	0.050047	0.050067	0.050089
Elacestrant	0.023923	0.027922	0.023105	0.023145	0.023185
Toremifene	0.053872	0.038494	0.052046	0.052056	0.052067
Fulvestrant	0.119436	0.066552	0.109928	0.109580	0.109237
Megestrol acetate	0.053243	0.039443	0.054764	0.054661	0.054559
Anastrozole	0.059358	0.034970	0.057962	0.057822	0.057685
Exemestane	0.026531	0.023178	0.023585	0.023602	0.023620
Letrozole	0.024103	0.022771	0.023585	0.023662	0.023739
Alpelisib	0.059538	0.042425	0.061160	0.060984	0.060810
Ixabepilone	0.039033	0.035512	0.037495	0.037461	0.037428
Abemaciclib	0.046947	0.042018	0.047009	0.046966	0.046923
Capivasertib	0.035615	0.032530	0.035017	0.035075	0.035133
Ribociclib	0.060618	0.045271	0.062119	0.062037	0.061958
Neratinib	0.059988	0.052862	0.070994	0.070906	0.070820
Olaparib	0.041011	0.036868	0.041173	0.041159	0.041147
$I^{+}$	0.119436	0.066552	0.023105	0.109580	0.109237
$I^{-}$	0.023923	0.022771	0.109928	0.023145	0.023185

The weighted normalized matrix is $x_{ij}=w_{j}^{*}\cdot t_{ij},\;j=1,\ldots ,n$.

Step 4: Determine the optimal positive and negative answers (Table 11). Determining how an option differs from the ideal one, which is defined as

$$I^{+}=\left\{x_{i}^{+},\ldots ,x_{j}^{+}\right\}=\left(max\;\left(or\;min\right)\;X_{ij}\backslash \;j\in J\right),$$

$$I^{-}=\left\{x_{i}^{-},\ldots ,x_{j}^{-}\right\}=\left(max\;\left(or\;min\right)\;X_{ij}\backslash \;j\in J\right).$$

Step 5: To obtain the separation measure, use the n-dimensional Euclidean distance (Table 13). The best response is set apart from each other by

$$D_{i}^{+}=\sqrt{\overset{n}{\underset{j=1}{\sum }}{\left(X_{ij}-I_{j}^{+}\right)}^{2}},$$

$$D_{i}^{-}=\sqrt{\overset{n}{\underset{j=1}{\sum }}{\left(X_{ij}-I_{j}^{-}\right)}^{2}}.$$

**TABLE 13:** TOPSIS results for the selected drugs.

Drugs	$D_{i}^{+}$	$D_{i}^{-}$	$C_{i}^{*}$	TOPSIS rank
Tamoxifen	0.115021	0.077512	0.402591	8
Elacestrant	0.159659	0.086976	0.352650	13
Toremifene	0.111807	0.078514	0.412535	7
Fulvestrant	0.086823	0.160983	0.649633	1
Megestrol acetate	0.110112	0.078474	0.416120	6
Anastrozole	0.105633	0.080608	0.432817	5
Exemestane	0.158856	0.086386	0.352248	14
Letrozole	0.160304	0.086347	0.350078	15
Alpelisib	0.101611	0.082949	0.449441	4
Ixabepilone	0.134138	0.077749	0.366937	11
Abemaciclib	0.119296	0.077397	0.393491	9
Capivasertib	0.139168	0.078286	0.360012	12
Ribociclib	0.099650	0.084584	0.459109	3
Neratinib	0.094796	0.090952	0.489651	2
Olaparib	0.129134	0.076584	0.372276	10

Step 6: Determine $C_{i}^{*}$ for each replacement using the following procedure (Table 13):

$$C_{i}^{*}=\frac{D_{i}^{-}}{D_{i}^{+}+D_{i}^{-}},\;\left(0<C_{i}<1,i=1,\ldots n\right).$$

Step 7: Sort the alternatives according to the results found in Table 13.

### 3.6 Simple additive weighting for ranking drugs

In multicriteria decision-making, the Simple Additive Weighting (SAW) method is a fundamental and popular approach. The weighted normalized values of each criterion are added together to rank the alternatives. The approach is simple to use and provides clear result interpretation. The best alternative is the one that receives the highest overall score. In this approach, each criterion is given a suitable weight, and the weighted sum of the alternative normalized values is determined using the formula $S_{i}=\sum_{j=1}^{n}w_{j}*\cdot r_{ij}$ .

The alternative deemed most appropriate is the one with the greatest $S_{i}$ value. Table 14 displays the computed scores for each alternative acquired using the SAW technique for easy comparison and ranking.

**TABLE 14:** SAW results for the selected drugs.

Drugs	M1β(G)	M1*β(G)	M2β(G)	HMβ(G)	Fβ(G)	${\text{S}}_{i}$	SAW rank
Tamoxifen	0.432229	0.558045	0.455273	0.456904	0.458530	0.461005	8
Elacestrant	0.200301	0.419552	0.210182	0.211214	0.212242	0.228782	14
Toremifene	0.451054	0.578411	0.473455	0.475050	0.476639	0.479519	7
Fulvestrant	1.000000	1.000000	1.000000	1.000000	1.000000	0.999999	1
Megestrol acetate	0.445783	0.592668	0.498182	0.498821	0.499457	0.495747	6
Anastrozole	0.496988	0.525458	0.527273	0.527672	0.528070	0.520452	5
Exemestane	0.222139	0.348269	0.214545	0.215387	0.216226	0.229722	13
Letrozole	0.201807	0.342159	0.214545	0.215932	0.217313	0.224858	15
Alpelisib	0.498494	0.637475	0.556364	0.556523	0.556682	0.551070	4
Ixabepilone	0.326807	0.533605	0.341091	0.341862	0.342629	0.356887	11
Abemaciclib	0.393072	0.631365	0.427636	0.428597	0.429555	0.440010	9
Capivasertib	0.298193	0.488798	0.318545	0.320087	0.321623	0.331330	12
Ribociclib	0.507530	0.680244	0.565091	0.566140	0.567186	0.563744	3
Neratinib	0.502259	0.794297	0.645818	0.647069	0.648316	0.628183	2
Olaparib	0.343373	0.553971	0.374545	0.375612	0.376675	0.385424	10

## 4 Conclusion

In this study, the molecular structures of 15 breast cancer drugs were converted into molecular graphs allowing for the successful use of chemical graph theory. $M_{\beta }$ -polynomials were used to compute resolving topological indices, which yielded important structural details. Overall, the study indicates that ORCT, ORAT, and WS demonstrate moderate predictive accuracy, while Caco-2 and SP show weak-to-moderate correlations with the resolving topological indices. The TOPSIS and SAW multicriteria decision-making techniques were then used to assess and rank these drugs’ ADMET characteristics. A comparison of the two methods, presented in Figure 13, shows that the obtained ranks are generally consistent. Additionally, the ranking is consistent with published clinical research (Dubiański, 2022). For instance, it has been demonstrated that the combination of ribociclib with fulvestrant improves survival and slows the course of advanced ER-Positive (Estrogen Receptor Positive)/HER2-Negative (Human Epidermal Growth Factor Receptor 2 Negative) breast cancer. This demonstrates the usefulness of the suggested ranking method. Future studies can build on this technique by including bigger datasets, entropy-based topological indices, and new physicochemical or pharmacological properties. Overall, the proposed approach provides a structured framework for evaluating and ranking drugs, although its predictive potential has limitations and should be taken with caution.

**FIGURE 13:**
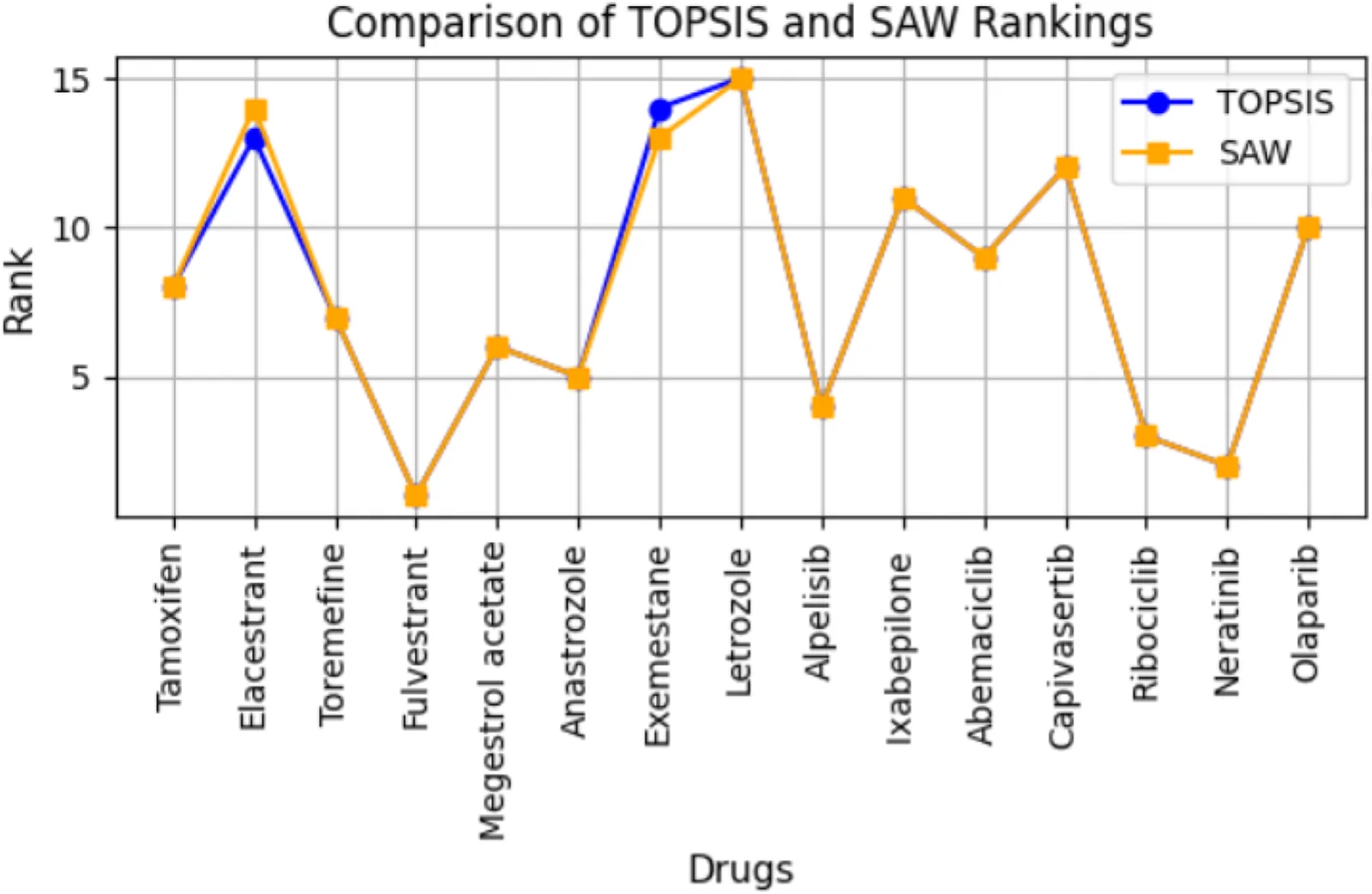
Comparison of TOPSIS and SAW rankings.

## 5 Limitations

Although the current work indicates the use of entropy-based weighting for ranking ADMET properties and resolving topological indices, several limitations must be addressed. First, the study is fully based on computer-based data, including topological indices and selected ADMET properties, and it does not include any experimental or clinical testing. Therefore, the results should be seen as computational predictions, not final proof of drug performance. Second, the entropy technique allocates weights only using data variability and ignores the biological or therapeutic significance of pharmacological factors. In actual drug development, qualities such as toxicity and bioavailability are frequently more important than others, independent of statistical fluctuation. As a result, the derived rankings may not accurately represent real-world therapeutic priority.

Finally, the study is limited to 15 FDA-approved breast cancer drugs and a small set of ADMET features. The brute-force method for determining resolving sets and metric dimensions works well for molecular graphs with less than 60 carbon atoms, but it becomes time-consuming as the graph size increases, restricting its use to larger molecular systems. Future research should include expert knowledge or biologically informed weighting techniques to increase the applicability and reliability of the results.

## Data Availability

All custom computer code used to generate the results is available in the GitHub repository at https://github.com/P16-GIT/Resolving-set.git
